# Supplementary figures and images for: An application of PCR-RFLP species identification assay for environmental DNA detection
Source: PeerJ. 2019 Oct 3;7:e7597. doi: 10.7717/peerj.7597 (PMC6778664; doi:10.7717/peerj.7597)

Figure S1: PCR-RFLP and gel electrophoresis of field experiment (A)

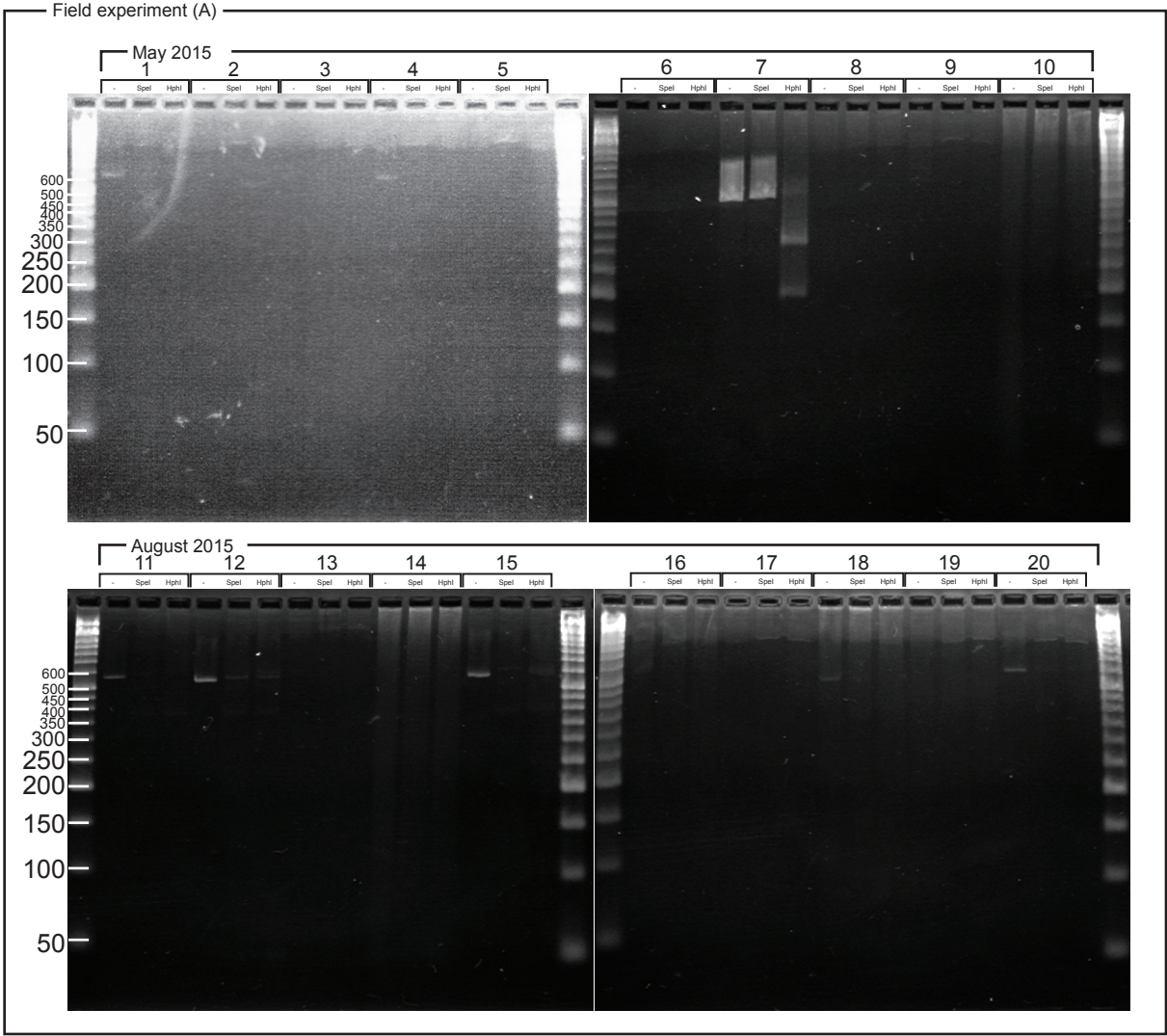

Supplement: Supplemental Information 1 [file peerj-07-7597-s001.pdf]

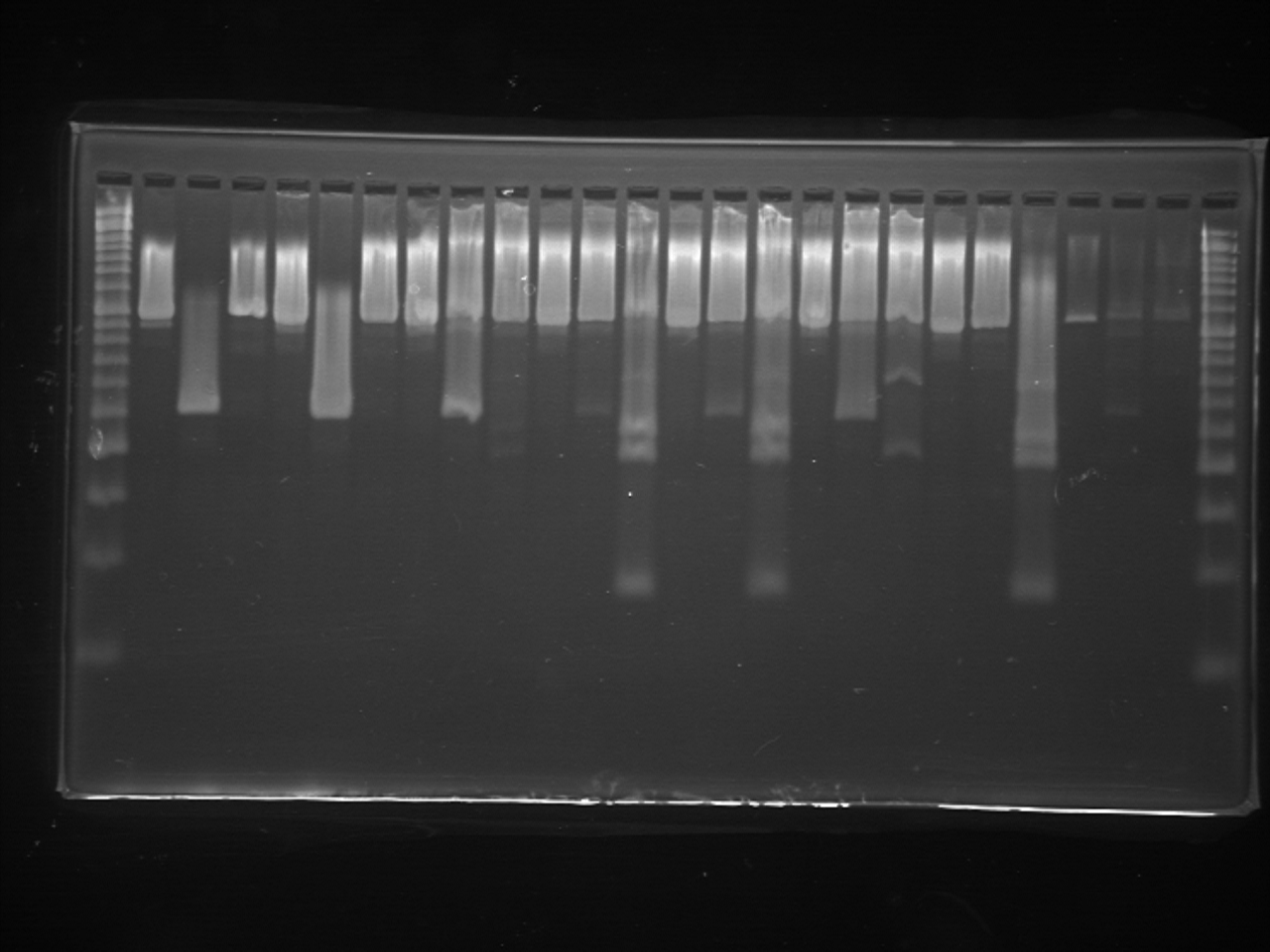

Supplement: Supplemental Information 3 [file peerj-07-7597-s003.bmp]

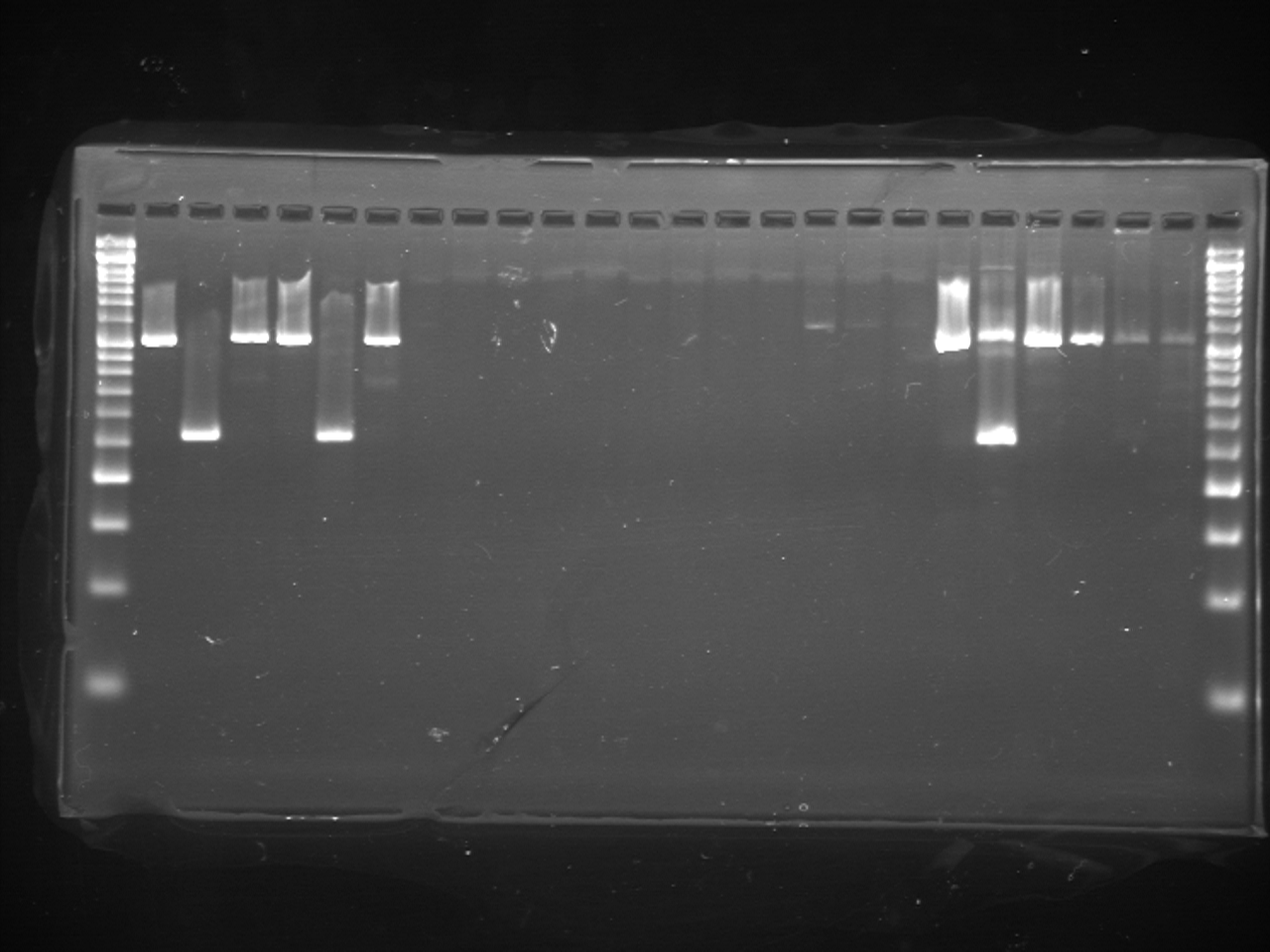

Supplement: Supplemental Information 4 [file peerj-07-7597-s004.bmp]

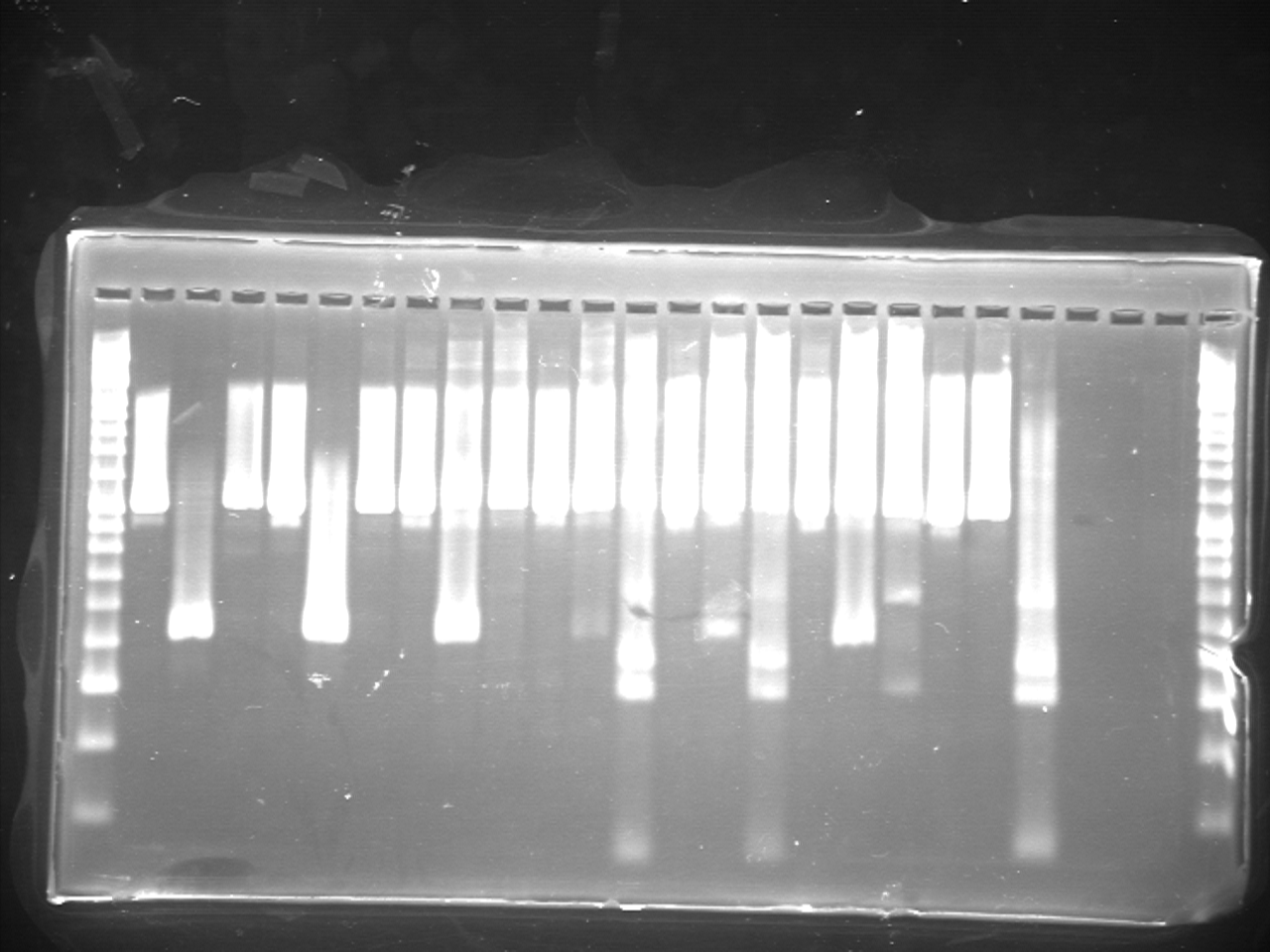

Supplement: Supplemental Information 5 [file peerj-07-7597-s005.bmp]

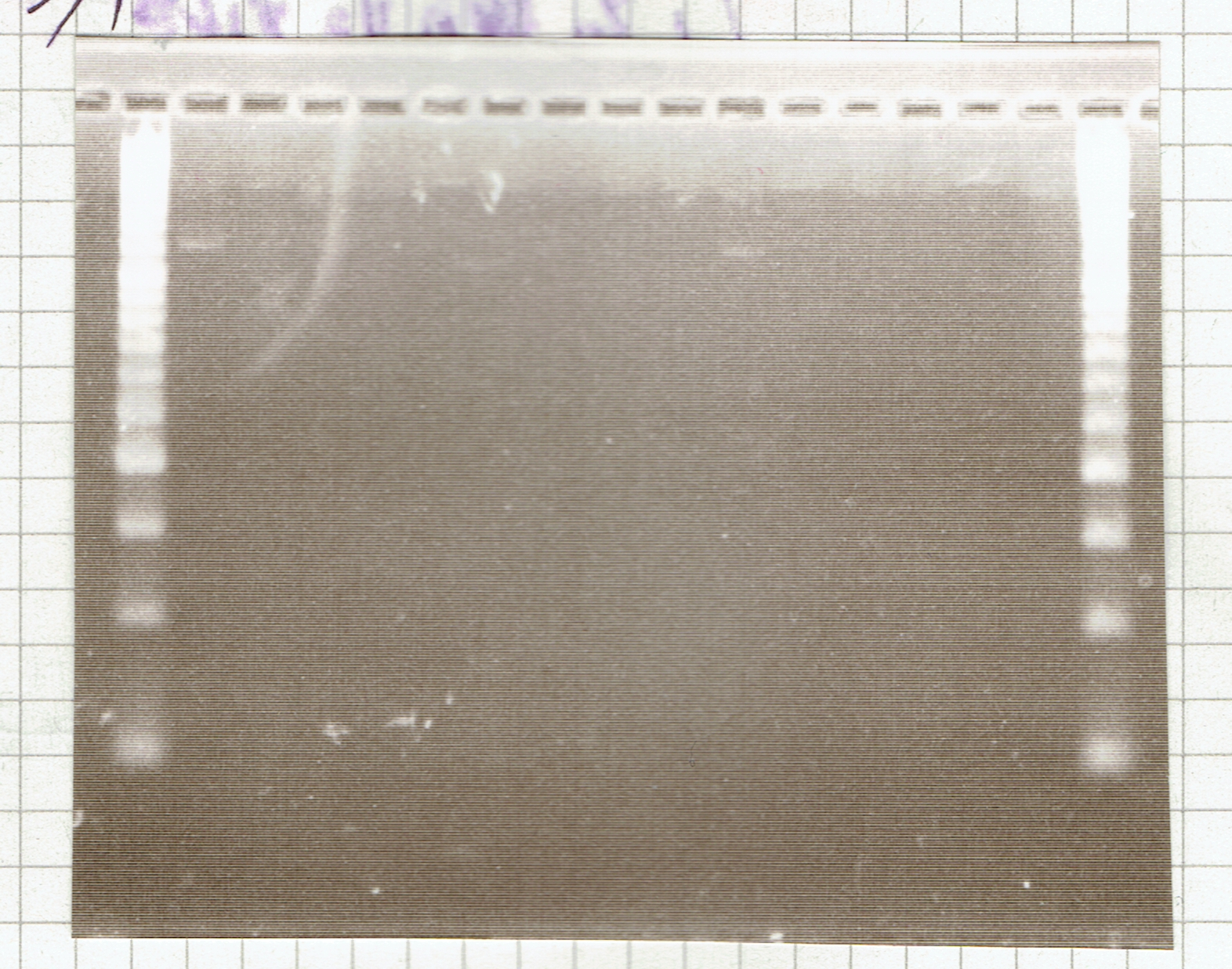

Supplement: Supplemental Information 6 [file peerj-07-7597-s006.bmp]

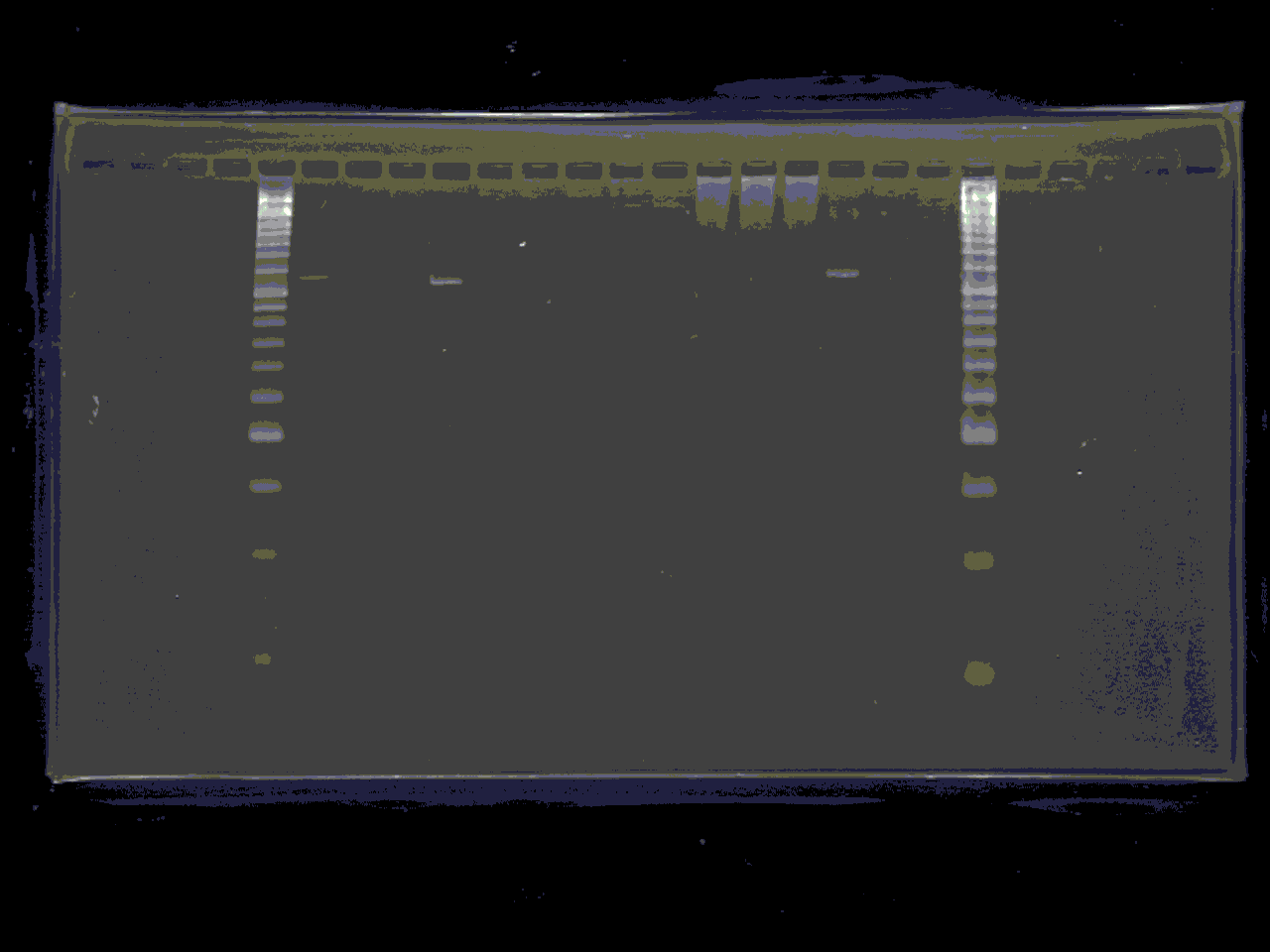

Supplement: Supplemental Information 7 [file peerj-07-7597-s007.bmp]

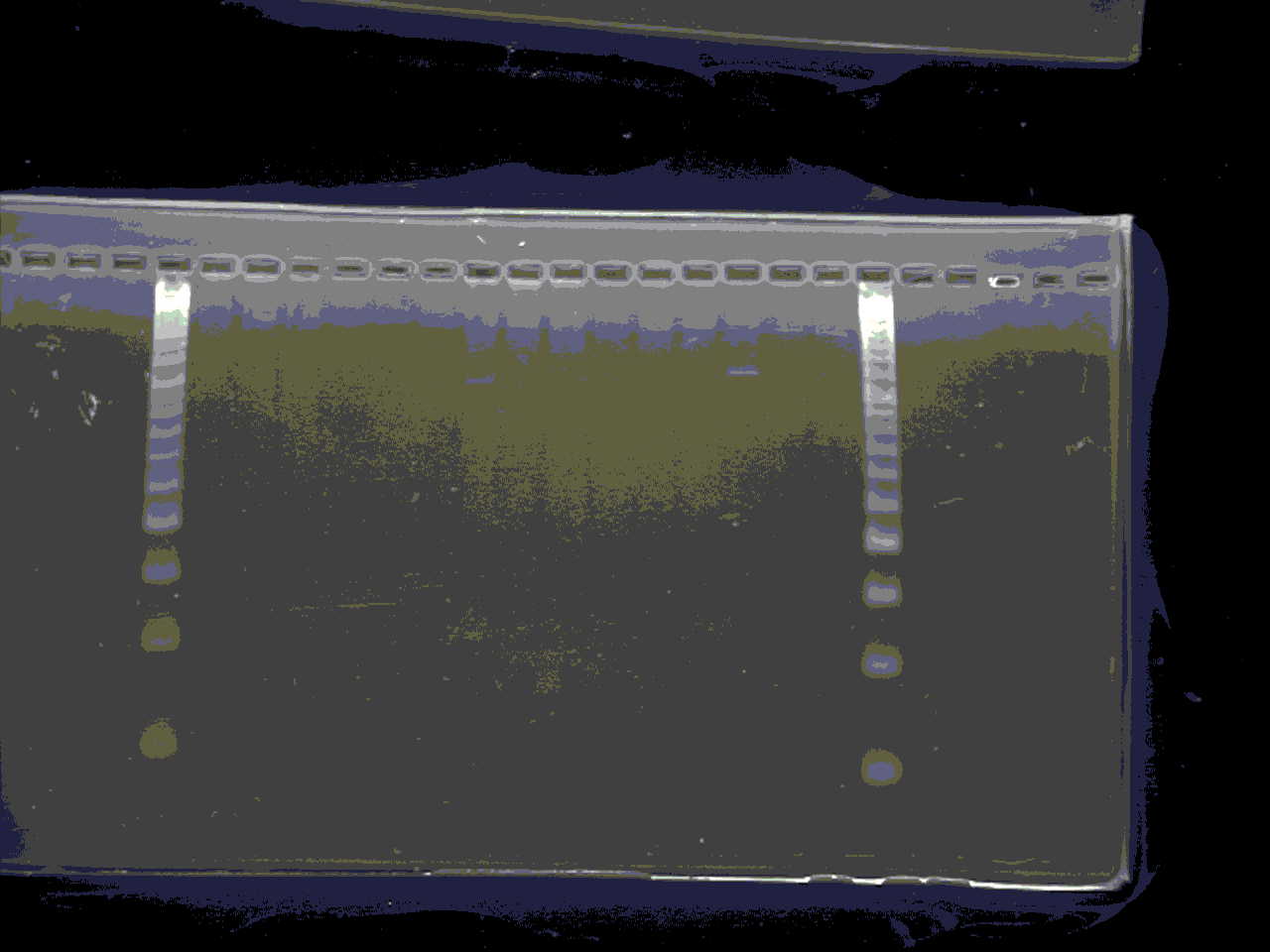

Supplement: Supplemental Information 8 [file peerj-07-7597-s008.bmp]
